# Quality determination and the repair of poor quality spots in array experiments

**DOI:** 10.1186/1471-2105-6-234

**Published:** 2005-09-26

**Authors:** Brian DM Tom, Walter R Gilks, Elizabeth T Brooke-Powell, James W Ajioka

**Affiliations:** 1 Medical Research Council – Biostatistics Unit, Institute of Public Health, Robinson Way, Cambridge CB2 2SR, UK; 2 Department of Pathology, University of Cambridge, Cambridge CB2 1QP, UK; 3 Washington University, School of Medicine, Department of Molecular Microbiology, 660 S. Euclid Avenue, CB 8230, St. Louis, MO 63110, USA

## Abstract

**Background:**

A common feature of microarray experiments is the occurence of missing gene expression data. These missing values occur for a variety of reasons, in particular, because of the filtering of poor quality spots and the removal of undefined values when a logarithmic transformation is applied to negative background-corrected intensities. The efficiency and power of an analysis performed can be substantially reduced by having an incomplete matrix of gene intensities. Additionally, most statistical methods require a complete intensity matrix. Furthermore, biases may be introduced into analyses through missing information on some genes. Thus methods for appropriately replacing (imputing) missing data and/or weighting poor quality spots are required.

**Results:**

We present a likelihood-based method for imputing missing data or weighting poor quality spots that requires a number of biological or technical replicates. This likelihood-based approach assumes that the data for a given spot arising from each channel of a two-dye (two-channel) cDNA microarray comparison experiment independently come from a three-component mixture distribution – the parameters of which are estimated through use of a constrained E-M algorithm. Posterior probabilities of belonging to each component of the mixture distributions are calculated and used to decide whether imputation is required. These posterior probabilities may also be used to construct quality weights that can down-weight poor quality spots in any analysis performed afterwards. The approach is illustrated using data obtained from an experiment to observe gene expression changes with 24 hr paclitaxel (Taxol ^®^) treatment on a human cervical cancer derived cell line (HeLa).

**Conclusion:**

As the quality of microarray experiments affect downstream processes, it is important to have a reliable and automatic method of identifying poor quality spots and arrays. We propose a method of identifying poor quality spots, and suggest a method of repairing the arrays by either imputation or assigning quality weights to the spots. This repaired data set would be less biased and can be analysed using any of the appropriate statistical methods found in the microarray literature.

## Background

Until fairly recently, little research has concentrated on design and quality issues in microarray studies. Instead, most research has been devoted to developing methods for analysis. Indeed this is understandable as the final endpoint of any study is to answer the biological questions of interest. However, the importance of experimental design and quality control cannot be over-emphasised, as experiments that have not been designed based on sound principles are more likely to produce poor quality data, which in turn affects all downstream processes (image analysis, transformation, normalization, statistical analysis) and thus lead to unreliable or misleading results. Work by researchers such as [1-3], etc. have attempted to address the deficit in the area of experimental design. However, far fewer researchers have tackled the issue of quality control, some exceptions being the work done by [4-7].

In microarray studies, researchers are often interested in comparing two or more mRNA samples either to determine which genes are differentially expressed or to detect different subtypes [8,9]. The approach adopted in analysing the data depends not only on the question(s) of interest, but also on the quality of the microarray data. Statistical methods such as cluster analysis may be quite sensitive to the process of filtering out poor quality expression data (i.e. missing data), whilst other methods such as principal component analysis and singular value decomposition cannot be used when missing data are present in the matrix of gene expressions [10].

Missing data in microarray experiments occur for a number of different reasons, including the quality of the clone preparation and of the mRNA, image corruption, the printing process, the presence of dust or scratches on the array, saturation, incomplete hybridization etc. In the pre-processing of the raw intensity data (i.e. image extraction and analysis, normalization and transformation), quality assessment of individual spots plays an integral part. Poor quality spots, either flagged up manually or through quality measures provided by the image extraction software or developed in the literature, are usually filtered out so as to prevent bias in results. Additionally, negative background-corrected intensities become undefined if a logarithmic transformation of the data is used, and must be removed. Thus this filtering process may potentially lead to a substantial amount of missing data, in particular for cDNA experiments when a relative measure of gene expression is of interest and therefore valid intensity measurements in both the red (Cy5) and green (Cy3) channels are required. Consequently, compared to the situation when there is complete data, the efficiency and power of any analysis performed on the filtered data may be substantially reduced.

Furthermore, if data "missingness" is due to the removal of less reactive spots or intensities below some pre-defined (often arbitrary) limit of detection, then any analysis performed on the incomplete data set may be subject to unwanted bias. The incomplete data set may not be representative of the complete data set, as the filtering process may be highly selective in the types of genes affected. Thus the missingness is informative, and ways of appropriately addressing the missingness are required to obtain more relevant and meaningful results. In particular, replacing undefined logarithmic data with zeros or shifting the data by a positive constant, although common approaches, may not be valid.

It is generally agreed that experimental replication (biological replicates) and repeated measurements (technical or analytic replicates) are fundamental requirements in the experimental design of microarray experiments, as they are critical for reliably distinguishing noise from other sources of variation (important or otherwise), thereby increasing the reliability and consistency of results obtained [2,11]. Furthermore, as we will show, replication may serve the additional purpose of allowing a more thorough determination of spot quality and open the way to handling poor quality spots through imputation or weighting. In this paper we describe a modelling approach to "repair" microarray data sets that, by the filtering of poor quality or negative intensities, have missing data.

## Results

### Example

We apply our method (see Methods section) to background uncorrected intensity data obtained from an experiment performed to observe gene expression changes with 24 hr paclitaxel (Taxol ^®^) treatment on a human cervical cancer derived cell line (Hela). In this experiment the cells were treated with l0 nM paclitaxel at 50% confluency and left for 24 hours prior to RNA extraction. Six independent RNA biological replicates (six flasks each extracted individually) were created for treated samples. These were compared using identically configured 4.5 K human cDNA microarrays (HGMP, Hinxton) with a common reference DMSO-treated sample used as control. Each array had 8448 probes spotted.

To calibrate and stabilize the variance of the background uncorrected intensity data, the inverse hyperbolic sine (arsinh) transformation of [12] was applied using the "vsn" package in R (Bioconductor Project). The RI (or MA) plots (figures not shown) of the transformed data for the six arrays showed that the transformation stabilized the variance reasonably well for most of them. However, Array 2 had rather different scale and offset parameters than the other five arrays. This may be indicative of a "problem" with Array 2, although not necessarily, as the arsinh transformation may have corrected for any systematic differences between the six arrays. However, as will be seen later, Array 2 was observed to have the largest proportion of spots identified as being of poor quality amongst the six arrays.

The results of fitting the mixture model described in the Methods section to our data are presented in the tables below. The parameters *α *and *ε *were set to 3 and 0.01 respectively. Estimates of some of the location parameters, the variances and the component probabilities are shown in Table 1. The automated approach estimated that approximately 81% of the intensities in channel F635 (Cy5-channel) were of good quality, whilst 98% of the intensities in channel F532 (Cy3-channel) were estimated to be of good quality. Table 2 displays the number and proportion of poor quality (failures) spots identified by our proposed method under stochastic flagging. We observe that there are significantly more poor quality data predicted for the F635 channel than the F532 channel. Further, we observe that Array 2 has the highest proportion of failures in both channels amongst the six arrays (54% and 3% in the F635 and F532 channels respectively), thus confirming our earlier observation. This array is partly responsible for only 81% of the intensities overall in F635 being of good quality. When Array 2 is removed and the quality assessment is repeated the failure proportions for each channel are more evenly spread across the five remaining arrays (data not shown) and the percentage of good quality intensities in channel F635 improves to 90%, whilst in channel F532 it becomes 97%. Interestingly, of the double failures occurring in Array 2 through to Array 6, the percentage of unreliably low poor quality spots in both channels were approximately 5%, 83%, 60%, 33% and 87% respectively.

**Table 1:** Parameter estimates from the model. Estimated parameters from the mixture model used to assess quality. The *μ*_1*ck *_estimates are not shown, since there are a large number of genes.

F635 Channel (c = 1)		F532 Channel (c = 2)
parameter	estimate	estimate

*μ*_0*c*_	-0.37	-0.72
*μ*_2*c*_	0.99	1.45
	0.11	0.23
	0.11	0.23
	0.22	0.47
*π*_*c*_(0)	0.013	0.010
*π*_*c*_(1)	0.808	0.976
*π*_*c*_(2)	0.178	0.013

**Table 2:** Failures in the six arrays. Numbers and proportions predicted as failing in each channel for the six arrays.

	F635 Channel Failures		F532 Channel Failures		Double Channel Failures	
Array	Failures	% Total	Failures	% Total	Failures	% Total

1	30	0.4	8	0.1	0	0
2	4539	54	225	3	204	2
3	1345	16	43	0.5	29	0.3
4	1180	14	13	0.2	5	0.1
5	191	2	13	0.2	9	0.1
6	444	5	58	0.7	23	0.3

Total	7729		360		270	

It was reassuring that the majority of spots identified by eye as of poor quality (data not shown) prior to undertaking the automatic quality assessment analysis were also identified through the proposed method.

**Figure 1:**
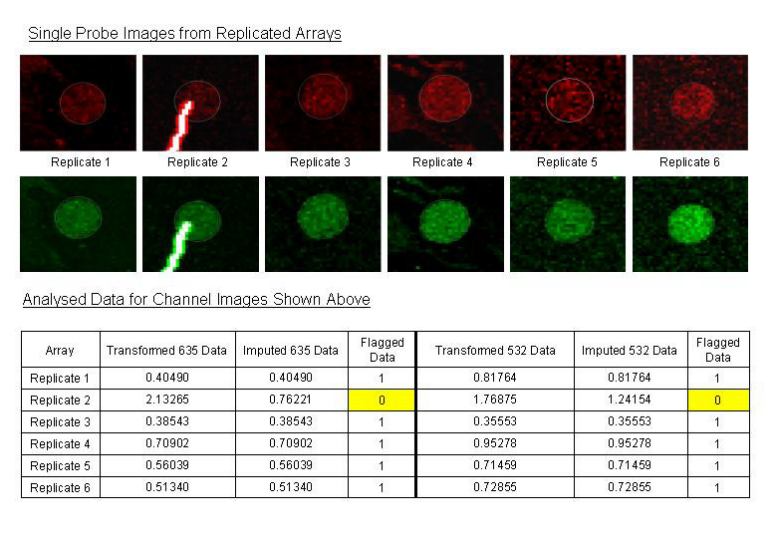
**Spot quality identification**. Spot quality identification. The spot on Array 2 has been identified as being of poor quality in both channels due to dust on the slide at that position.

An example of the type of spots clearly identified by eye and also identified through use of the model as being of poor quality is shown in Figure 1. Here dust on the slide at this spot position has caused an obvious saturated flash in both channels of Array 2. By imputation under stochastic flagging, we have replaced these poor intensities with more reasonable intensity measurements that are comparable to the same spot on the other five arrays. In addition to identifying these clear "outlier" spots, note that more subtle differences between replicates that could not be picked up manually by "eyeing" the data are easily detected with the automated approach.

**Figure 2:**
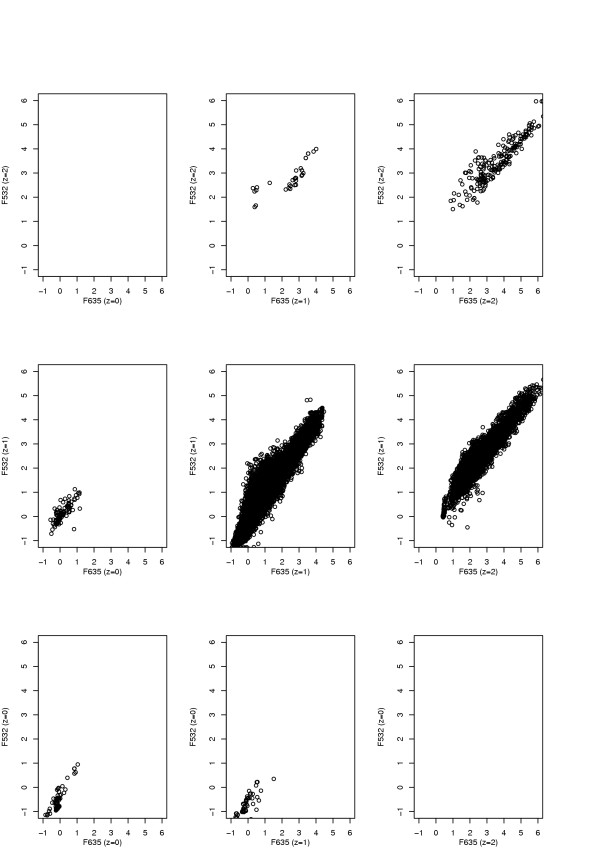
**Bivariate scatter plot of transformed data**. The bivariate scatter distribution of the transformed intensity data, *y*_*ikc*_. *z *= 0, 1, 2 correspond to the poor component with unreliably low intensities, the good component and the poor component with unreliably high intensities.

The resulting bivariate scatter plot of the transformed data using our model (under stochastic flagging) to predict the quality labels is shown in Figure 2. Most of the data fall in the middle box, whilst the majority of data are in the upper right four panels. This indicates that most of the poor quality data were of the unreliably high variety. Note the strong positive correlation between *y*_*ik*1 _and *y*_*ik*2 _in each cell of Figure 2. This clearly reflects underlying correlations in the *μ*_1*k*1 _and *μ*_1*k*2_, for example. Note that this does not conflict with the conditional independence assumption for our model, which as stated in the Methods section, concerns the residuals *r*_*ik*1 _and *r*_*ik*2 _Note also that overlap in intensity measurements across panels is allowable, as each spot intensity measurement for a particular probe is made relative to the replicate spot intensities for that probe across arrays.

**Figure 3:**
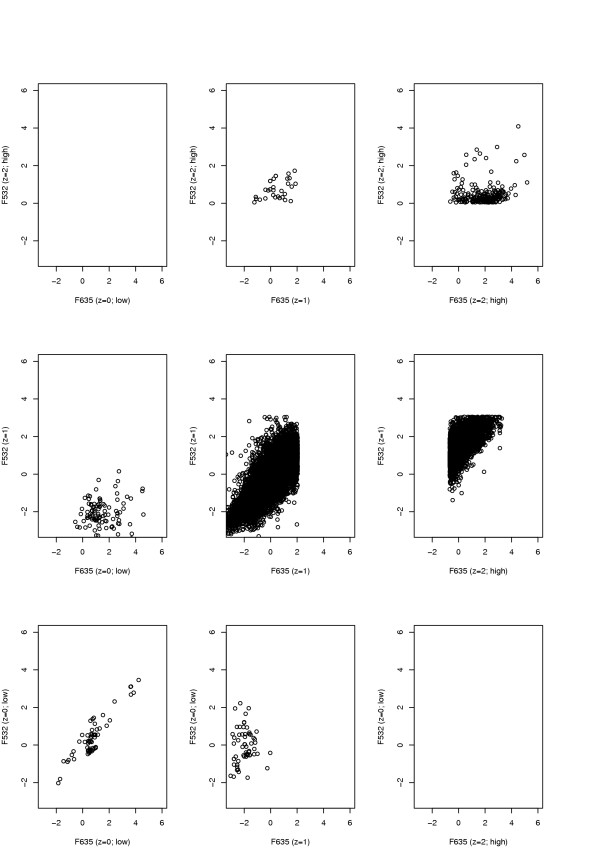
**Bivariate scatter plot of residuals**. The bivariate scatter distribution of the residuals, *r*_*ikc*_. *z *= 0, 1, 2 correspond to the poor component with unreliably low intensities, the good component and the poor component with unreliably high intensities.

**Figure 4:**
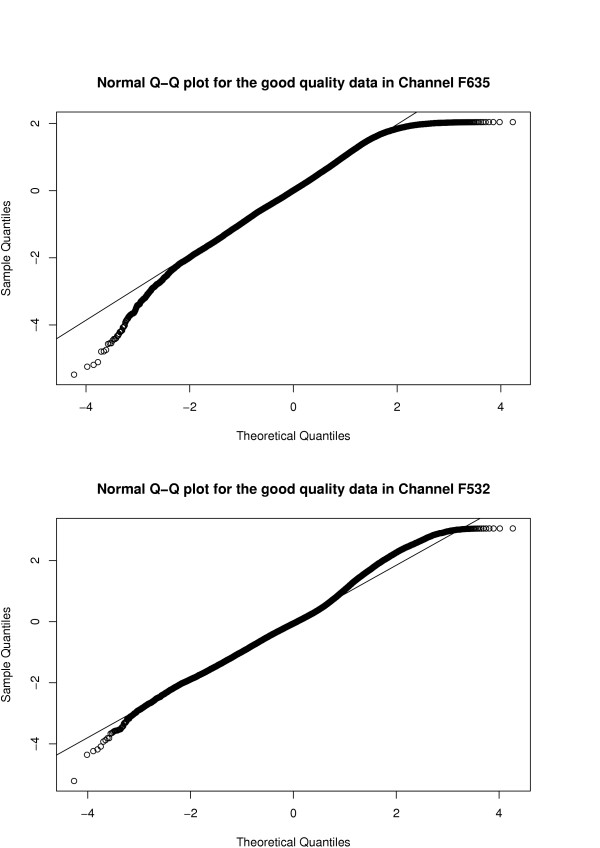
**Quantile-Quantile plots**. Quantile-Quantile plot for spots predicted as good quality in each channel.

Figure 3 shows the resulting bivariate residual scatter plot using the predicted quality labels. Note that because of the way spots were classified into quality categories, we observe an apparent vertical or horizontal "boundary" in some of the panels where the residuals cannot go beyond (e.g. in Panels 5 and 6). Quantile-quantile plots for the residuals predicted as good quality are shown in Figure 4. Note that there appears to be reasonable fit between the observed residuals and the expected residuals from a standard normal distribution, except in the tails. The deviations in the tails are partly due to the way spots are classified into quality categories (boundary effect described above), partly due to the constraints placed on the parameters and also due to the influence of Array 2 (especially in Channel F635).

Here the strong positive correlations that were observed in Figure 2 is now only apparent in the middle panel and the lower left-hand panel. We have experimented with more elaborate models that take account of this dependence, but the results were not substantially changed. We therefore prefer to stay with the model described in the Methods section, as it is simpler.

In the microarray literature, a number of approaches have been developed for missing value estimation or imputation. These approaches range from the simple "replace missing entries with zeroes" and row-average approaches to K-nearest neighbourhood (KNN) approach and its variants such as the sequential KNN (SKNN) approach [10,13], to singular value decomposition (SVD) and Bayesian principal component analysis (BPCA) methods [10,14], to least square, regression and maximum likelihood approaches [15-18]. The most popular of these is the KNN approach, which was shown by [10] to outperform the row-average and SVD approaches. However, in terms of root mean square error (RMSE), it was shown not to perform as well as the more complex and time consuming approaches [14-18] that have been recently proposed or its variant SKNN approach, which was shown to have improved accuracy in estimation of missing data with high computational speed. Additionally, an imputation approach using Gaussian mixture clustering [19] has been developed and found to be more accurate than the SVD and KNN approaches. This imputation method is similar in spirit to our mixture modelling approach.

We have compared the imputation part of our approach with the KNN and SKNN approaches for missing data estimation. We assume that the imputed data set constructed from our example above is a "true" data set. From this true data set, we randomly select 1000 spots, and perturb their F635 (Cy5) intensities by any independent Gaussian random variable with mean ± 1 and variance 2. These probes may then indicate possible "high" or "low" poor quality spots depending on how extreme the applied perturbations. From this newly pertubated data set, we apply our method to flag poor quality spots. Of the 1000 pertubed spots, those flagged were then filtered and the KNN and SKNN methods were applied to repair the data for these spots. The root mean square errors (RMSEs) of the KNN and SKNN methods were then calculated for these flagged spots and compared to the RMSE obtained from our mixture model assuming that the flagged intensity data were replaced by the appropriate "good quality" spots' mean parameters. We repeated the above ten times and the average root mean square errors were calculated for the three imputation approaches.

It was found that on average 539 of the 1000 randomly chosen spots were flagged by the quality assessment step of our method. The RMSE for our mixture model approach was 0.475, which was substantially smaller than the RMSEs for the KNN and SKNN approaches, which were 0.733 and 0.727 respectively.

## Discussion

We have demonstrated an alternative approach to assessing the spot quality in cDNA microarray experimentation. The method requires replicate arrays in order to assess whether a spot signal is a true signal or not. Its strength lies in the use of information found within and, also importantly, between arrays. Thus we are able to separate different components of variability found in microarray experiments. This then allows us to be able to identify subtle problems that cannot be detected by considering each array separately, as well as the more obvious problems such as dust and comet tails. Data that appear to be good when assessed within an array, need not be reliable when assessed against corresponding data from replicate arrays. Thus replication increases the power of detecting poor and good quality spots and therefore reduce the false positive and false negative rates.

The data from replicate arrays, in its raw or background corrected form, may in general not be comparable because of the need for separate calibration and normalization of the arrays. We chose to use the inverse hyperbolic sine transformation of [12] to do the necessary calibration and normalization. This transformation was shown to be very effective and robust when compared to alternative transformations discussed in the literature.

Our approach has the additional advantage of not filtering out data but instead imputing new data to replace the spots (in either or both channels) identified as being unreliable. Of course, it is necessary to acknowledge that these imputed data are not real data and therefore suitable measures must be taken to account for the resulting uncertainty in further analyses using these repaired data. We advocate the use of multiple imputation as a way to avoid spuriously precise results. Furthermore our method performed favourably to the KNN and SKNN missing data/imputation approaches, with the added generality/advantage that it not only repairs poor quality spots but identifies them.

Alternatively instead of imputing new data, our approach can be used to assign weights to each spot. These quality weights can then be used under various strategies to down-weight spots thought to be of uncertain quality in downstream microarray processes, such as normalization and statistical analyses. Some researchers have advocated filtering of unreliable spots to avoid biasing results. We believe that the filtering of spots does not necessarily remove biases, but may actually introduce bias if the data filtering process is informative. That is, for example, if the filtering process is highly selective in removing certain types of genes and therefore the resulting filtered data set will not be representative of the true data. As our approach is dependent on having replicates, it is natural to ask how many replicates are required. However, the number of replicates required depends on a number of factors, such as the type of microarray experiment to be performed (i.e. design and analysis issues), the reliability of the experimental system used (i.e. taking into account quality issues), the cost, etc. In the example above, five or six replicates appeared to be a reasonable number. However in other types of experiments a larger number of replicates may be required. [20] provide a useful discussion regarding this question.

## Conclusion

As the quality of microarray experiments affect downstream processes, it is essential to have a reliable and automatic method of flagging and then repairing poor quality spots. We have proposed a mixture model method to accomplish this two-step process of identification and imputation, and thereby producing a repaired/complete data set which is less biased than before.

## Methods

### Quality assessment and the mixture model

At present, microarray spot quality is assessed via two approaches. The first is based on the physical characteristics of the spot. That is, the noise, the size, shape and position of each spot, and the development of a composite quality score that reflects these features [5,20]. The second is based on assessing the quality of spots through consistency (in terms of whether or not a spot is expressed) of replicated results from a number of similar arrays [4,7]. Below, we describe an alternative approach to quality control that addresses the identification of poor quality data and how to replace them. We discuss this approach in the case of two-channel cDNA microarrays, but believe that the method can be extended to other types of array experiments (oligonucleotide, Affymetrix chips etc.), with minor modifications. In a two-channel cDNA microarray experiment, two samples of mRNA are labelled with different fluorescent dyes, commonly Cy3 (green dye) and Cy5 (red dye), and co-hybridized onto a microarray of thousands of known cDNA clones (probes) immobilised on glass supports. The image data obtained from the experiment for each spot on the array are in the form of (Cy3, Cy5) spot or target-intensity pairs, representing the expression levels of the corresponding genes in the two mRNA samples. For each channel (Cy3 or Cy5) in each spot, the observed intensity may be thought of as one of three quality types:

**Type 0 **poor quality, where the observed intensity is unreliably low in relation to other replicates;

**Type 1 **good quality, where the observed intensity is valid; and

**Type 2 **poor quality, where the observed intensity is unreliably high in relation to other replicates.

Fundamentally, we maintain that omitting any of the above categories of poor quality data can lead to serious biases. Therefore we do not filter out poor quality data, but instead we model the distribution of the data in each channel conditionally independently (given the spot) as a three-component mixture distribution. For this approach to be useful, replication is required. A fuller description of the model we propose is outlined below.

Furthermore, pre-processing (normalization and transformation) of the replicate arrays is required to make them comparable to each other. We have chosen to use the inverse hyberbolic sine (arsinh) transformation of [12] (also see [21]), instead of the logarithmic transformation, to transform and normalize the data. This transformation can be written mathematically as



where *a*_*ic *_and *b*_*ic *_are array-dependent (i.e. *i*th array) and channel-specific (i.e. *c*th channel) parameters,  is the original intensity reading for the *k*th spot in the *c*th channel of the *i*th array and *y*_*ikc *_is the corresponding transformed value. This transformation is used for three reasons. Firstly, it is defined over the entire real line and thus the problem of obtaining undefined values with logarithmic transformations is avoided. Secondly, this transformation has been shown to be more effective at stabilizing the variance over the entire range of intensities for various types of microarray experiments, thus removing any relationship between the variance of the spot intensities with their means. Also this transformation behaves similarly to the logarithmic transformation for large intensities. Finally, this transformation, due to its specific array-dependent parameters, can robustly and independently calibrate (normalize) the data from each microarray. [22] have investigated, via simulation, the usefulness of different transformations and found that, in a variety of situations, the arsinh transformation performs well in terms of straightening the curvature seen in RI (MA) plots and in stabilizing the variance of the microarray data. Additionally, they found it to be one of the transformations providing the greatest increase in power (compared to the logarithmic transformation) to distinguish differential genes from non-differential genes, with the detectable fold change being only slightly reduced. Of course, variance stabilization following the use of the arsinh transformation nevertheless needs to be checked in each case by, for example, looking at the MA plot.

We assume that the observed data on this transformed scale can be modelled independently for each channel, conditional on the spot's true mean *μ*_1*kc*_, as a mixture of three normal distributions corresponding to the three quality types defined above. Essentially, therefore, our assumption of conditional independence concerns the independence of the *residual noise *in each observation (see equation (3)), not of the observations themselves. The assumption of normality is in keeping with how this transformation was developed, and fits in with the standard methodological assumptions made when fitting microarray data. Note that, at the outset, we do not know to which of the three components each observed intensity belongs. Our task is to infer this from the *replicate *data. Denoting an observed (Cy3, Cy5) transformed target-intensity pair for the *k*th spot in the *i*th array by the bivariate response *y*_*ik *_= (*y*_*ik*1_, *y*_*ik*2_), then the mixture probability density, , is the product of the mixture probability densities of the Cy3 intensity,  and of the Cy5 intensity, . For conciseness, we denote these three densities as *f*(*y*_*ik*1_, *y*_*ik*2_), *f*_1_(*y*_*ik*1_) and *f*_2_(*y*_*ik*2_) respectively, where we suppress the dependence on the parameters. Mathematically, we write



where



and where *f*_*c*_(*y*_*ikc*_|*z*_*ikc*_) is the conditional distribution of *y*_*ikc*_, given that *y*_*ikc*_, is of type *Z*_*ikc *_and *π*_*c*_(*z*) is the prior probability that an observation from channel *c *is of type *z*, where *z *= 0,1, or 2.

Now the conditional distributions *f*_*c*_(*y*_*ikc*_|*z*_*ikc*_) for the Cy3 and Cy5-spot intensities are assumed to have the same form. They are described as shown below



where the means *μ*_1*k*1 _and *μ*_1*k*2 _depend on *k*, but all the variances:  and , and the remaining means: *μ*_01_, *μ*_02_, *μ*_21 _and *μ*_22 _do not. However, to prevent non-identifiability the following constraints on *μ*_0*c*_, *μ*_2*c*_, and *π*_*c*_(*z*), are specified: *μ*_0*c *_≤ *min*(*μ*_1*kc*_: ∀*k*), *μ*_2*c *_≥ *ασ*_1*c *_for *c *= 1, 2 where *α *is a user-specified positive parameter, and *π*_*c*_(*z*) ≥ *ε *for *c *= 1, 2 and *z *= 0, 1, 2, where *ε *is a user-specified parameter in the range (0, 1/3). For example, *α *= 3 indicates that the unrelaibly high mean should be three standard deviation (*σ*_1*c*_) away from the true signal mean, *μ*_1*kc*_. An *ε *of 0.01 would indicate that the proportion of poor quality spots in our arrays will not be less than 2% of the total number of probes. Additionally, the following constraints on the variance parameters are added:  and  for *c *= 1, 2. That is, the measurement error associated with Type 1 data should not be greater than the variability attached to poor quality data.

We believe that the above conditional distributions have biological plausibility. Spot intensities of Type 0 are affected by either incomplete hybridization or suboptimal incorporation of the dye or strongly affected by high background noise. Our model (2.1) asserts that an observed intensity of Type 0 does not contain any information about the target at that spot. Therefore we assume that all spot intensities of Type 0 in a particular channel will have a common mean and also a common variance.

Most of the data should be of Type 1 in well performed experiments. Our model (2.2) asserts that an observed intensity of Type 1 reliably reflects biologically meaningful information about the target at that spot. We have assumed that the spot intensities for each probe (across replicate arrays) in a channel will have a probe-specific mean signal (representing gene-specific levels of up or down regulation), but a variance which is common across probes. The assumption of common variance appears a reasonable one to make especially after the transformation and normalization step is performed on the data.

Spot intensities of Type 2 are affected by dust or scratches, etc.. Our model (2.3) asserts that such intensities reflect a biologically meaningful (true) signal, *μ*_1*kc*_, plus a bias, *μ*_2*c*_, due to unwanted signal effects caused by the dust or scratches. Note that the discrimination between these three types can only be achieved through replicate data. Replication allows us to assess the reproducibility/reliability of the observed spot intensities.

Under our proposed model, we are able to construct residuals of the form



which will allow us to assess the appropriateness of our model assumptions, through, for example, quantile-quantile plots (i.e. Q-Q plots) and other graphical methods. Note that these graphical methods may only be useful after the components where spots belong are identified.

Assuming that we have *N *technical or biological replicates (i.e. *N *arrays), then the observed joint probability density (or observed likelihood) is a product of the mixture distribution (1) over the *K *spots and the *N *replicates. That is, the observed likelihood, *L*, takes the form



Our aim is to identify for each spot its most likely component. Where an observation is predicted to be of Type 0 or Type 2, we aim to replace it with an imputed value. To achieve this goal, we adopt the strategy below, where Points 3a and 3b represent two alternative and independent versions of assigning intensities to type.

1. Estimate the mean, variance and component probability parameters, (*μ*, *σ*^2^, *π*), through maximum likelihood, using the likelihood (4), subject to the constraints being satisfied.

2. Calculate the channel-specific posterior probabilities, *θ*_*ikcl *_= *p*(*z*_*ikc *_= *l*|*y*_*ikc*_, *μ*, *σ*^2^, *π*), for belonging to each of the three components for each spot in each replicate, when given the observed intensity data and the estimates obtained in Step 1 above. These posterior probabilities are given by



3. The mixture component for each channel can be assigned to each spot in each array in either of two independent ways:

a. *Deterministic Flagging: *Assign intensity *y*_*ikc *_to the Type *l *having maximum posterior probability *θ*_*ikcl*_; or

b. *Stochastic Flagging: *Assign intensity *y*_*ikc *_to the Type *l*, where *l *is sampled with probability *θ*_*ikcl *_for *l *= 0, 1, 2.

4. Where the Type *l *assigned to *y*_*ikc *_is not 1, *y*_*ikc *_is replaced by an imputed value sampled independently from (2.2).

Alternatively instead of following Points 3 and 4 of the above strategy, the user can assign a weight *w*_*ik *_= *θ*_*ik*1*l*_*θ*_*ik*2*l' *_to each spot. These weights can then be used in downstream analyses with already developed software packages (e.g. LIMMA).

These strategies are implemented through use of an Expectation-Maximization (E-M) algorithm [23,24]. In our implementation, the algorithm has been modified to take into account the constraints imposed on the parameters to avoid non-identifiability. Also, to avoid the potential problem of the unboundedness of the log-likelihood, the maximum likelihood estimate of the variance parameters have been modified as shown below.



where the σ*^2^s are the modified (weighted) versions of *σ*^2^s, *β *= *M/K *- 1 = *N *- 1,  for *c *= 1, 2, and *M *is the total number of observations. This modification of the variance terms is motivated by Bayesian arguments. The constraints placed on the original variance parameters also apply to these modified variances.

Note that our approach allows us to borrow strength across probes/spots in order to estimate most of the parameters in our model, the exception being the estimation of the *μ*_1*k*1 _and *μ*_1*k*2 _parameters which rely only on the observations from the replicates of the *k*th spot.

A variety of different stopping strategies may be applied to determine when the parameter estimates in our model have converged. These range from looking at the relative changes in the log-likelihood from one iteration of the E-M algorithm to the next, to multivariately assessing the changes in all the parameter estimates from one iteration to the next, through the use of an appropriately defined distance metric. We prefer to use the change in the log-likelihood to determine convergence. However, since for the constrained E-M algorithm the log-likelihood need not increase at each iteration, we instead stop the algorithm if at the current iteration the log-likelihood obtained is larger than at previous iterations and when the change between this maximum current value of the log-likelihood and the previous maximum value of the log-likelihood (over the previous iterations) is smaller than a pre-specified convergence value, or when a pre-specified number of iterations have been completed. Some fine tuning of the convergence value may be required, as also may be the case for the choice of initial values for the parameters.

### Repairing the microarray data-set via imputation

Following the above rules in Points 3 (either 3a or 3b) and 4 above, we obtain a repaired complete intensity data set. If a single imputation of the data set is all that is required, then either deterministic flagging (Point 3a) or stochastic flagging (Point 3b) can be used.

Note that a drawback of the single imputation approach is that the imputed values are treated as if known, and therefore in future analyses using this singly repaired data set, no acknowledgement will be made of the uncertainty that results from imputing the values. That is, these analyses will ignore the variability due to imputation [24] and estimates obtained may be spuriously over-precise. Thus many researchers working in the area of missing data, recommend the use of multiple imputation over single imputation. Thus, it may be preferable to generate a few multiple repaired data sets (Point 4) using, say "stochastic flagging" as in Point 3b above to identify spots which require imputation. Subsequent analyses may then be performed on each repaired data set. Results may then be compared between these data sets to ensure that any conclusions are consistent across these data sets. Alternatively, more formal methods can be used [25]. In the example described earlier, we generate just a single imputation although our algorithm may be used to generate multiple imputations.

## Authors' contributions

BDMT and WRG developed the methodology for this paper, analysed the data, and drafted the paper. ETBP and JWA designed and carried out the microarray experiments, provided the data, and contributed to the drafting of the paper. All authors read and approved the final manuscript.
